# Rates of Trauma Exposure and Posttraumatic Stress in a Pediatric Digital Mental Health Intervention: Retrospective Analysis of Associations With Anxiety and Depressive Symptom Improvement Over Time

**DOI:** 10.2196/55560

**Published:** 2024-02-27

**Authors:** Darian Lawrence-Sidebottom, Landry Goodgame Huffman, Aislinn Brenna Beam, Rachael Guerra, Amit Parikh, Monika Roots, Jennifer Huberty

**Affiliations:** 1 Bend Health Inc Madison, WI United States; 2 FitMinded Inc LLC Phoenix, AZ United States

**Keywords:** collaborative care model, telehealth, childhood trauma, DMHI, digital health, mental health, telemedicine, trauma, traumatic, pediatric, pediatrics, paediatric, paediatrics, child, children, youth, adolescent, adolescents, teen, teens, teenager, teenagers, retrospective, anxiety, depression, depressive, co-occurring, comorbid, comorbidity, comorbidities, association, associations, correlation, correlations, correlate

## Abstract

**Background:**

More than 2 out of 3 children and adolescents in the United States experience trauma by the age of 16 years. Exposure to trauma in early life is linked to a range of negative mental health outcomes throughout the lifespan, particularly co-occurring symptoms of posttraumatic stress (PTS), anxiety, and depression. There has been an increasing uptake of digital mental health interventions (DMHIs) among youths, particularly for anxiety and depression. However, little is known regarding the incidence of trauma exposure and PTS symptoms among youths participating in DMHIs and whether PTS symptoms impact anxiety and depressive symptom treatment response. Moreover, it is unclear whether participation in a DMHI for anxiety and depressive symptoms is associated with secondary effects on PTS symptoms among trauma-exposed youths.

**Objective:**

This study aims to use retrospective data from youths participating in a DMHI to (1) characterize rates of trauma, PTS, and comorbid anxiety and depressive symptoms; (2) determine whether trauma exposure and elevated PTS symptoms impact the improvement of comorbid anxiety and depressive symptoms throughout participation in care; and (3) determine whether participation in a non–posttraumatic DMHI is linked to reductions in PTS symptoms.

**Methods:**

This study was conducted using retrospective data from members (children ages 6 to 12 years) involved in a pediatric collaborative care DMHI. Participating caregivers reported their children’s trauma exposure. PTS, anxiety, and depressive symptom severity were measured monthly using validated assessments.

**Results:**

Among eligible participants (n=966), 30.2% (n=292) reported at least 1 traumatic event. Of those with trauma exposure and elevated symptoms of PTS (n=119), 73% (n=87) exhibited elevated anxiety symptoms and 50% (n=59) exhibited elevated depressive symptoms. Compared to children with no trauma, children with elevated PTS symptoms showed smaller reductions per month in anxiety but not depressive symptoms (anxiety: *F*_2,287_=26.11; *P*<.001). PTS symptoms also decreased significantly throughout care, with 96% (n=79) of participants showing symptom reductions.

**Conclusions:**

This study provides preliminary evidence for the frequency of trauma exposure and comorbid psychiatric symptoms, as well as variations in treatment response between trauma-exposed and nontrauma-exposed youths, among participants in a pediatric collaborative care DMHI. Youths with traumatic experiences may show increased psychiatric comorbidities and slower treatment responses than their peers with no history of trauma. These findings deliver compelling evidence that collaborative care DMHIs may be well-suited to address mental health symptoms in children with a history of trauma while also highlighting the critical need to assess symptoms of PTS in children seeking treatment.

## Introduction

More than two-thirds of children have experienced trauma, such as abuse, neglect, natural disasters, and sudden loss of a loved one, by the age of 16 years [1,2]. These rates have been exacerbated by the recent COVID-19 pandemic and associated lockdowns, which appear to have caused a significant increase in child maltreatment globally [3-6]. During the first year of the pandemic, more than 11% of US adolescents reported physical abuse and 55% reported emotional abuse—2- and 3-fold increases compared to prepandemic rates [7,8]. The far-reaching and pervasive effects of childhood trauma are well documented. Those who experience trauma, particularly in childhood and adolescence [9], are at increased risk for a number of maladaptive mental and physical health outcomes throughout the lifespan [10,11] including posttraumatic stress disorder (PTSD). PTSD and symptoms of posttraumatic stress (PTS) develop as a result of a traumatic event and include reexperiencing (eg, flashbacks or memories of the event), avoidance of reminders and feelings related to the event, and elevated arousal and alterations in cognition and mood (eg, negative emotions and feelings, blame, and isolation [12]). Recent estimates suggest that 16% of children and adolescents who experience trauma go on to develop PTSD, although symptom severity is often dependent on age and gender, as well as type, duration, and severity of the trauma experienced [13]. Those with trauma are not only at risk for developing PTSD but also a number of mental health difficulties, particularly anxiety and depression [14]. Indeed, PTS, depression, and anxiety share common symptoms, etiologies, and effective treatment modalities such as cognitive behavioral therapy (CBT) [15,16].

Youths with traumatic experiences are significantly more likely to receive mental health care from a variety of sources, including primary care physicians, therapists, psychiatrists, school counselors, and social workers [17]. With shortages of in-person mental health providers and rates of pediatric mental health disorders increasing, traditional modalities of mental health care are becoming steadily more overburdened, expensive, and inaccessible. These issues of accessibility paired with the lockdowns of the COVID-19 pandemic catalyzed widespread uptake of digital mental health interventions (DMHIs) or those facilitated by technologies such as computers and smartphones. Although a number of DMHIs are available for the treatment of pediatric PTSD [18], these interventions and associated research are limited in significant ways. First, no research has been done to characterize the rates of trauma among youths participating in DMHIs for comorbid symptoms such as anxiety and depression, which are some of the most prevalent mental health disorders among youths. Indeed, most youths who receive mental health services do so for anxiety and depressive symptoms [19-21]. Given the etiological overlap among PTS, depression, and anxiety, there is a high likelihood that many youths with PTS symptoms would experience secondary benefits when receiving mental health care for anxiety and depression. Second, there is little understanding of how traumatic experiences and PTS symptoms impact the treatment response of anxiety and depressive symptoms for youths participating in a DMHI. By exploring these 2 lines of research, pediatric DMHIs will be better equipped to adapt their care programs and modalities to the needs of users with traumatic experiences and posttraumatic symptoms.

The collaborative care model (CoCM), in which primary care providers partner with behavioral care managers (BCMs) and psychiatrists to coordinate patient-centered and measurement-based care, is widely considered the best practice for pediatric mental health care [22]. Researchers have argued that the CoCM, with its use of regular symptom measurement and individualized care, confers better outcomes, particularly for those with trauma who are exhibiting complex and comorbid symptoms of PTS, depression, and anxiety [23,24]. Early evidence indicates that DMHIs using the CoCM are associated with improvements in pediatric mental health problems, including anxiety and depression [10,25,26]. However, no research has been done to understand the use and effectiveness of collaborative care DMHIs for anxiety and depression among trauma-exposed youths.

Therefore, the purpose of this study was to use retrospective data from youths participating in a collaborative care DMHI to (1) characterize rates of trauma, PTS, and comorbid anxiety and depressive symptoms; (2) determine whether trauma exposure and elevated PTS symptoms impact improvement of comorbid anxiety and depressive symptoms throughout participation in care; and (3) determine whether participation in a non–posttraumatic DMHI is linked to reductions in PTS symptoms.

## Methods

### Participants

Bend Health Inc members aged 6 to 12 years (at baseline, before care started) were eligible for inclusion in the study if they (1) had their first coaching or therapy session with Bend between January 1, 2023, and October 1, 2023 (9 months), and (2) completed the trauma assessment before beginning care (N=979). To more specifically assess symptom outcomes for members receiving care for mental health symptoms other than PTS, members who participated in the trauma care program were excluded from all analyses (n=13, 1.3%). Thus, the final sample included 966 members.

### Ethical Considerations

Study procedures were approved by the Biomedical Research Alliance of New York (Study 23-12-034-1374). All participants provided informed consent to their data being used for research purposes upon enrollment, and all data were anonymized and deidentified prior to analysis. Bend Health Inc members were not compensated for their participation in this retrospective research.

### Treatment

Treatment with Bend Health Inc has been described previously [25]. Bend Health Inc is a DMHI for youths that uses the CoCM to implement a whole-family approach, involving caregivers in treatment. Each member is assigned a behavioral care manager (BCM) who oversees and manages the child’s individual treatment plan and works with primary care providers, psychiatrists, therapists, and coaches to determine the correct treatment plan for each member. The member then meets regularly with either a licensed therapist or a coach, depending on the type and severity of mental health symptoms the member is experiencing. To specifically target a particular symptom domain (eg, anxiety symptoms), children are assigned a care program (by their BCM) based on their symptom severity and care goals. All care programs are designed to be developmentally appropriate for the age of the member, and the primary care programs (eg, anxiety, depression, and attention-deficit/hyperactivity disorder [ADHD]) are intended to take approximately 12 weeks to complete. During sessions, coaches and therapists provide behavioral care that is informed by the components of the care program. The informational contents of all care programs are also available in a digital platform for members and their caregivers to access between sessions (asynchronously). Once a month, caregivers are asked to complete questionnaires regarding their child’s symptoms, including PTS, anxiety, and depressive symptoms.

Therapy at Bend Health Inc provides diagnostic clarity, addresses complicated history of trauma and problematic behaviors, and provides clinical treatment for mental health disorders. Coaching provides behavior change tools and improvements in self-efficacy using evidence-based best practices. When appropriate, members’ care can escalate to include both coaching and therapy for the treatment of more severe symptoms. Both coaching and therapy at Bend Health Inc are based on CBT, behavioral activation, motivational interviewing, caregiver training, and mindfulness-based practices. Depending on symptom needs and care plan, members may also meet with a psychiatrist at enrollment and throughout care for additional symptoms and medication management.

### Assessments

At enrollment into care with Bend Health Inc, caregivers are asked to report their child’s demographic information, including date of birth, sex, gender, and race or ethnicity. The response options for sex are “male,” “female,” and “other.” The response options for gender are “male,” “female,” “transgender,” “nonbinary,” and “other.” From January 1, 2023, to May 26, 2023, only 1 race or ethnicity response could be selected, and the options were “White,” “Black or African American,” “American Indian or Alaska Native,” “Asian,” “Hispanic or Latino,” and “Other.” From May 26, 2023, to October 1, 2023, multiple race or ethnicity responses could be selected, and the options were “White,” “Black or African American,” “American Indian or Alaska Native,” “Chinese,” “Vietnamese,” “Native Hawaiian,” “Filipino,” “Korean,” “Japanese,” “Chamorro,” “Other Asian,” “Other Pacific Islander,” “Some other race or multi-racial,” “Mexican,” “Mexican American,” “Chicano,” “Puerto Rican,” “Cuban,” and “Another Hispanic, Latino, or Spanish origin.”

To assess children’s mental health symptoms during the enrollment process, caregivers first respond to screener questions. When elevated symptoms are flagged by the responses to the screeners, caregivers are then prompted to complete fully validated assessments. To screen for PTS, caregivers are asked the question: “Has your child ever experienced a traumatic event?” If the response to this question is “Yes,” caregivers are then asked to report the nature and timing of the child’s most distressing event, and they also complete the entire Child PTSD Symptom Scale (for the Diagnostic and Statistical Manual of Mental Disorders, Fifth Edition; CPSS-V) validated questionnaire [27]. To assess the nature of the traumatic event, caregivers are asked to describe their child’s most distressing event in a free textbox. To assess the timing of the traumatic event, caregivers are asked “How long has it been since that event occurred?” with the following response options: “1-30 days,” “1-3 months,” “3-6 months,” “6-12 months,” “1-2 years,” “2-4 years,” and “4+ years.” The CPSS-V consists of 20 items, in which caregivers are asked to report how often their child exhibits behaviors consistent with PTS, such as “trying not to think about it [the distressing event] or have feelings about it” and “trouble having good feelings.” Responses to these items are made on a 5-item Likert-type scale, with responses ranging from “not at all” (score=0) to “6 or more times a week/almost always” (score=4).

Screener questions for anxiety and depressive symptoms are taken from the Diagnostic and Statistical Manual of Mental Disorders, Fifth Edition (DSM-5) cross-cutting measure, which asks caregivers to report the frequency (in the last 2 weeks) that their child exhibits behaviors associated with anxiety and depression [28]. There are 3 anxiety symptom screener questions and 2 depressive symptom screener questions. Responses to the anxiety and depressive symptom screeners are made on a 5-item Likert-type scale with responses ranging from “not at all” (score=0) to “nearly every day” (score=4). If a caregiver responds to any anxiety or depressive screener question with “several days” (score=2) or more frequently, they are prompted to complete the PROMIS (Patient-Reported Outcomes Measurement Information System) anxiety assessment or PROMIS depressive assessment, respectively [29]. The PROMIS anxiety assessment includes 10 questions about common anxiety symptoms (eg, feeling worried). The PROMIS depression assessment includes 11 questions about common depressive symptoms (eg, feeling lonely). For both PROMIS assessments, caregivers report the frequency of their child’s behaviors or feelings in the last 7 days, with responses on a 5-item Likert-type scale ranging from “never” (score=1) to “almost always” (score=5). Caregivers were prompted to complete mental health symptom screeners and assessments within the web-based portal every month after enrollment to track mental health symptom severity throughout care.

### Statistical Methods

Responses to all items from the CPSS-V were aggregated for a total PTS score of 0 to 80. Using standardized criteria, PTS symptom severity was determined based on CPSS-V scoring norms [27], which are as follows: minimal (scores: 0 to 10), mild (score: 11 to 20), moderate (score: 21 to 40), severe (score: 41 to 60), and very severe (score: 61 to 80). Responses to the items from the anxiety and depression PROMIS assessments were aggregated for a total anxiety score of 10 to 50 and a total depressive symptom score of 11 to 55, respectively. Then, total PROMIS scores were converted to t-scores using standardized criteria [30]. Anxiety and depressive symptom severity were then determined based on t-scores as follows: none to slight (t-score <55), mild (t-score 55-59.9), moderate (t-score 60-69.9), and severe (t-score ≥70). For PTS, anxiety, and depressive symptoms, symptom severity of moderate, severe, or very severe was considered “elevated.”

Standard descriptive statistics—including percent, mean (SD), and median (IQR)—are used throughout the “Results” section, as appropriate. For all analyses (outlined in detail below), between-group comparisons for categorical variables were performed using chi-square tests, and comparisons for continuous variables were performed using 2-tailed Wilcoxon signed rank tests or 2-tailed *t* tests, as appropriate based on data distribution (determined by Shapiro-Wilk test). Where between-group comparisons could not be performed given a small representation within a category of interest, only the descriptive statistics are reported.

### PTS Symptoms

**Figure 1:**
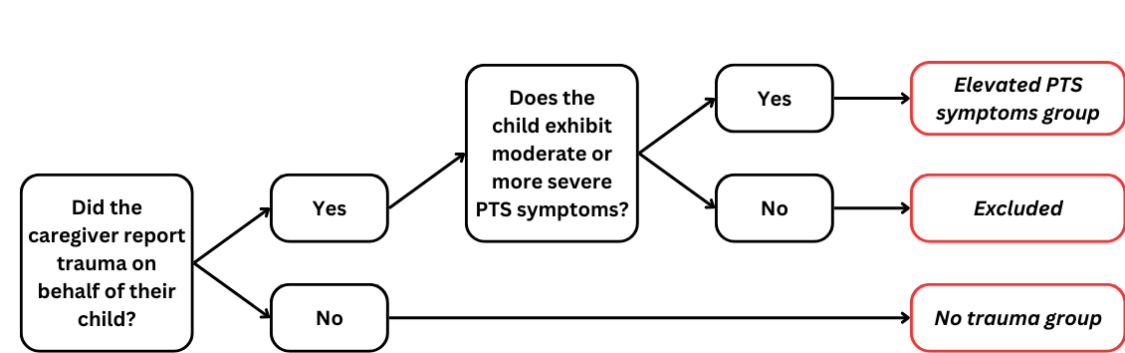
Flowchart delineating study group formation and exclusion criteria. PTS: posttraumatic stress.

For all members included in the study (n=966), the rates of reported trauma at baseline (last assessment before care started) were described. For members that had a traumatic event, CPSS-V scores, PTS symptom severity, and the timing of the traumatic event were reported. Members with no traumatic event were included in the no trauma group. Members with both a traumatic event and CPSS-V scores indicating moderate or greater PTS symptoms were included in the elevated PTS symptoms group. Members with a traumatic event and nonelevated PTS symptoms were not included in the primary analyses, and thus all further analyses were applied only to members in the “no trauma” and “elevated PTS symptoms” groups (Figure 1).

### Member Characteristics and Care Use

Member characteristics and care use patterns were reported for each group. The following member characteristics were assessed: age in years (at baseline), sex (male, female, and nonbinary), gender conforming (conforming and nonconforming), ethnicity (Asian, Black or African American, Hispanic/Latino, White, and Other), and mental health diagnoses by type (anxiety disorder, depressive disorder, and ADHD). Date of birth was used to calculate age in years (at enrollment). If a member’s sex at birth and gender identity (reported at enrollment) were not identical, they were classified as gender nonconforming. Otherwise, members were classified as gender conforming. Details on the reporting of race or ethnicity are included in the Multimedia Appendix 1. Rates of elevated mental health symptoms at baseline (moderate or greater severity) were also assessed for anxiety and depressive symptoms. For care use patterns, the duration of care (months between the first session and the last session) and participation in coaching and therapy were reported only for members with at least 1 coaching or therapy session (106 excluded). Between-group comparisons were performed for the following variables of interest: age, female sex (yes or no), gender conformity (conforming or nonconforming), ethnicity (White or non-White), elevated mental health symptom (elevated or nonelevated; all types), months in care, and participation in therapy (yes or no).

### Mental Health Symptom Reduction

PTS symptom reduction was assessed for members in the “elevated PTS symptoms” group. Rates of members with symptom reduction were assessed for those with at least 1 coaching or therapy session, and who completed at least one symptom assessment after starting care (37 excluded). Symptom reduction was considered a decrease in score from baseline or screening out of the last assessment. Then, the amount of total change over the duration of care was assessed by delta CPSS-V score from baseline to the last *full* CPSS-V assessment (no screened-out assessments), and delta scores were compared to 0 using a Wilcoxon signed rank test to assess for a significant change in score. This analysis was performed on data from members with at least 1 coaching or therapy session, and at least 1 full symptom assessment after starting care (54 excluded). To determine whether PTS symptoms decreased over months in care, CPSS-V scores were assessed over months in care by a linear mixed effects model with a fixed effect of months in care and a random effect of member ID on the intercept. Potential covariates were added to this basic model, and if a potential covariate improved model fit (based on the likelihood ratio test [LRT]), it was included in the final model. Models with the addition of the following covariates were tested against the basic model: age (at baseline), sex (female vs nonfemale), and race or ethnicity (White vs non-White).

Anxiety and depressive symptoms were assessed over time in care and compared between groups. First, the rates of members with symptom reduction were assessed for members with elevated mental health symptoms at baseline, at least 1 coaching or therapy session, and at least 1 symptom assessment after starting care (anxiety symptoms: 576 excluded; depressive symptoms: 658 excluded). Symptom reduction was considered a decrease in t-score from baseline or screening-out of the last assessment. The rates of members with symptom reduction were compared between groups using chi-square tests. These analyses were performed on data from members with elevated mental health symptoms at baseline, at least 1 coaching or therapy session, and at least one full symptom assessment after starting care (anxiety symptoms: 626 excluded; depressive symptoms: 712 excluded). Then, the total change in t-score (delta t-score) from baseline to the last full assessment was compared between groups using a 2-tailed *t* test or Wilcoxon signed rank test, as determined based on sample distribution. Finally, the rate of anxiety and depressive symptom reduction was compared between groups using linear mixed effects analyses with a fixed effect of group, the interaction of the group with months (in care), and a random effect of the subject on the intercept.

For all linear mixed effects analyses, to ensure that the findings were not skewed by baseline assessments occurring very early before the start of care, members whose baseline assessment occurred greater than 1 month before the first coaching or therapy session were excluded (PTS symptoms: additional 6 excluded; anxiety symptoms: additional 10 excluded; depressive symptoms: additional 4 excluded). A single additional member (n=1) was excluded from the depressive symptom linear mixed effects analysis due to an outlier t-score. For between-group analyses of rates of symptom reduction and total change, we confirmed that each group took their last and *full* last assessments at approximately the same time in care by between-group Wilcoxon signed rank comparisons of assessment timing for each symptom domain.

## Results

### PTS Symptoms

Of the 966 members included in the study, 30.2% (n=292) had experienced a traumatic event and the remaining 69.8% (n=674) did not have a traumatic event. For members with a traumatic event, CPSS-V scores were a median of 18 (IQR 9-28), with scores ranging from 0 (minimal PTS symptom severity) to 61 (very severe PTS symptoms). Of members with a traumatic event, 86 (29.5%) had minimal symptoms, 87 (29.8%) had mild symptoms, 99 (33.9%) had moderate symptoms, 19 (6.5%) had severe symptoms, and 1 (0.3%) had very severe symptoms. As such, for members with a traumatic event, 59.2% (n=173) had nonelevated PTS symptoms and 40.8% (n=119) had elevated PTS symptoms.

While 63.7% (n=186) of all members with a traumatic event reported that the event occurred 1 or more years before baseline, the timing of the event varied (Table 1). Notably, the rate of children having experienced the event within the last 30 days was nearly twice as high for children with elevated PTS symptoms (n=14, 11.8%) versus nonelevated PTS symptoms (n=10, 5.8%). For all further analyses, 674 were included in the no trauma group (no trauma reported at baseline), 119 were included in the elevated PTS symptoms group (traumatic event and elevated PTS symptoms), and 173 were not included in further analyses (traumatic event and nonelevated PTS symptoms).

**Table 1:** Timing of trauma, respective to when it was reported at baseline, reported for members in the no trauma and elevated PTS symptoms groups.

Timing of trauma (prior to baseline)	Nonelevated PTS^a^ symptoms, n (%)	Elevated PTS symptoms, n (%)
Total	173 (59.2)	119 (40.8)
1 to 30 days	10 (5.8)	14 (11.8)
1 to 3 months	14 (8.1)	11 (9.2)
3 to 6 months	15 (8.7)	10 (8.4)
6 to 12 months	19 (11)	13 (10.9)
1 to 2 years	34 (19.7)	23 (19.3)
2 to 4 years	38 (22)	23 (19.3)
4 or more years	43 (24.9)	25 (21)

^a^PTS: posttraumatic stress.

### Member Characteristics

Children with elevated PTS symptoms were a median of 10 (IQR 9-11) years old, 56.3% (n=67) were female, and they were largely gender conforming (n=113, 95%; Table 2). In terms of their race or ethnicity, 44.5% (n=53) were “White” and 35.3% (n=42) were “Other” or multiracial. Compared to members with no trauma, members with elevated symptoms of PTS were older (*z*=–4.38; *P*<.001) and more predominantly female (χ^2^_1_=5.33; *P*=.02). Rates of elevated mental health symptoms at baseline were higher for children with elevated PTS symptoms compared to children with no trauma. Specifically, 72.6% (n=87) of children with elevated PTS symptoms had elevated anxiety symptoms compared to 33.9% (n=228) of children with no trauma (χ^2^_1_=63.55; *P*<.001). Approximately 1 in 2 children with elevated PTS symptoms also had elevated depressive symptoms (n=59, 49.6%) compared to 1 in 5 children with no trauma (n=135, 20%; χ^2^_1_=46.21; *P*<.001). Children with elevated symptoms of PTS had higher rates of participation in the anxiety care program (χ^2^_1_=7.75; *P*=.005) and depression care program (χ^2^_1_=3.79; *P*=.05; statistical trend) and lower rates of participation in the ADHD care program (χ^2^_1_=4.06; *P*=.04).

**Table 2:** Member characteristics reported for children in the no trauma and elevated PTS^a^ symptom groups. Between-group comparisons were performed with chi-square tests unless otherwise specified.

Member characteristics		No trauma (n=674), n (%)	Elevated PTS symptoms (n=119), n (%)	Between-group comparisons				
				Chi-square (*df*=1)		*P* value		
Age^b^ (years), median (IQR)		9 (7-11)	10 (9-11)	–4.38^c^		<.001^d^		
**Sex**					5.33		.02^d^	
	Female	299 (44.4)	67 (56.3)					
	Male	372 (55.2)	51 (42.9)					
	Other	3 (0.4)	1 (0.8)					
**Gender conformity**					0.21		.64	
	Conforming	629 (93.3)	113 (95)					
	Nonconforming	45 (6.7)	6 (5)					
**Ethnicity**					0.00		>.99	
	Asian	38 (5.6)	4 (3.4)					
	Black or African American	37 (5.5)	11 (9.2)					
	Hispanic or Latino	28 (4.2)	9 (7.6)					
	White	301 (44.7)	53 (44.5)					
	Other or multiracial	270 (40.1)	42 (35.3)					
**Elevated mental health symptom**								
	Anxiety	228 (33.9)	87 (72.6)	63.55		<.001^d^		
	Depressive	135 (20)	59 (49.6)	46.21		<.001^d^		
**Care program**								
	Anxiety	289 (42.9)	68 (57.1)	7.75		.005^d^		
	Depression	51 (7.6)	16 (13.4)	3.79		.05^e^		
	ADHD^f^	231 (34.3)	29 (24.4)	4.06		.04^d^		
	Behavior	86 (12.8)	13 (10.9)	0.17		.68		

^a^PTS: posttraumatic stress.

^b^Between-group comparisons were performed with a 2-tailed Wilcoxon signed rank test.

^c^*z* value for Wilcox signed-rank tests.

^d^*P* values<.05.

^e^*P* values<.10.

^f^ADHD: attention-deficit/hyperactivity disorder.

For members in the no trauma group who began coaching or therapy, they were in care for a median of 3.03 (IQR 1.63-4.50) months; 98.8% (n=479) were in coaching and 23.7% (n=115) were in therapy. For members in the elevated PTS symptoms group that began coaching or therapy, they were in care for a median of 3.03 (IQR 1.89-4.67) months, and 100% (n=102) were in coaching and 25.5% (n=26) were in therapy. The duration of care did not differ between groups (*z*=–1.08; *P*=.28), and the rates of members in therapy also did not differ between groups (χ^2^_1_=1.27; *P*=.26).

### Mental Health Symptom Reduction

The rates of reduction in anxiety and depressive symptoms from baseline to the last assessment did not differ between groups (anxiety: χ^2^_1_=1.33; *P*=.25 and depressive: χ^2^_1_=0.28; *P*=.59), with 84.3% (183/217) of all members exhibiting a reduction in anxiety symptom severity and 86.7% (117/135) of all members exhibiting a reduction in depressive symptom severity (Table 3). The amount of change (delta t-score) from baseline to the last *full* assessment also did not differ significantly between groups (anxiety: *z*=–0.35; *P*=.73 and depressive: t_51.23_=0.19; *P*=.85). Specifically, for all children, anxiety t-scores decreased by a median of 5 points (IQR –9 to 0) and depression t-scores decreased by a mean of 3.77 (SD 7.54) points. The number of months between baseline and the last assessment and baseline and the last full assessment did not differ between groups for anxiety and depressive symptoms (all *P*>.05).

**Table 3:** Change in anxiety and depressive symptoms from baseline. Rates of members with a reduction in symptom severity from baseline to their last assessment and the change in t-score from baseline to the last full assessment are reported for each group.

Mental health symptom					No trauma			Elevated PTS^a^ symptoms			Between-group comparisons^b^			
											Chi-square (*df*=1)			*P* value
**Percent with reduction in symptom severity (baseline to last assessment)**														
	Anxiety, n/N (%)			130/158 (82.3)			53/59 (89.8)			1.33			.25	
	Depressive, n/N (%)			80/94 (85)			37/41 (90)			0.28			.59	
**Delta t-score (baseline to last *full* assessment)**														
	**Anxiety^c^**										–0.35^d^			.73
		Median (IQR)	–5.0 (–9 to 0)			–5.0 (–8 to –2.5)								
		Participants, n	116			51								
	**Depressive^e^**										0.19^f^			.84
		Mean (SD)	–3.65 (7.70)			–4.00 (7.35)								
		Participants, n	55			26								

^a^PTS: posttraumatic stress.

^b^Between-group comparisons were performed with chi-square tests, unless otherwise specified.

^c^Between-group comparisons performed with a 2-tailed Wilcoxon signed rank test.

^d^*z* value for Wilcox signed rank test.

^e^Between-group comparison were performed with a 2-tailed *t* test.

^f^*t* value for *t* test.

In the linear mixed effects model of anxiety symptom severity, the main effect of the group was not statistically significant (*F*_1,155_=2.52; *P*=.11), indicating that the no trauma and elevated PTS symptoms groups did not differ in anxiety symptom severity. The interaction of the group with months (from care start) was significant (*F*_2,287_=26.11; *P*<.001), such that children with no trauma had larger anxiety symptom reduction per month (mean –1.23, SD 0.19) than children with elevated PTS symptoms (mean –1.12, SD 0.31). For depressive symptom severity, the main effect of the group was not statistically significant (*F*_1,74_=2.39; *P*=.13). The interaction of the group with months approached significance (*F*_2,112_=2.86; *P*=.06), as children with elevated PTS symptoms had slightly larger depressive symptom reduction per month (mean –0.65, SD 0.44) than children with no trauma (mean –0.64, SD 0.34).

**Figure 2:**
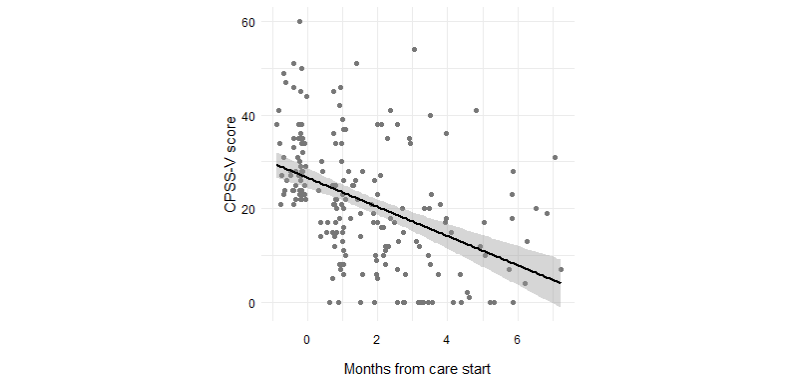
CPSS-V score over months in care for members with elevated PTS symptoms. CPSS: Child PTSD Symptom Scale; PTS: posttraumatic stress.

For those with elevated PTS symptoms, 96.3% (79/82) exhibited PTS symptom reduction from baseline to the last assessment, with the last assessment having a median of 2.33 (IQR 1.04-3.89) months after the start of care. For members who took the *full* CPSS-V after beginning care (n=65; median 2 months, IQR 1.03-3.50 after the start of care), CPSS-V scores decreased significantly from baseline (median change score –13 points, IQR –19 to –6; *z*=–6.35; *P*<.001). Results from the linear mixed effects model of PTS symptoms, which included a fixed effect of age (LRT: χ^2^_1_=3.97; *P*=.046) and female sex (LRT: χ^2^_1_=4.92, *P*=.03), showed that CPSS-V scores decreased significantly over months in care (*F*_1,140_=67.11; *P*<.001) by an estimated mean of 3.37 (SD 0.41) points per month (Figure 2). The main effects of age (*F*_1,54_=2.07; *P*=.16) and female sex (*F*_1,54_=1.42; *P*=.24) were not statistically significant.

## Discussion

### Principal Findings

The purpose of this study was to use retrospective data from youths participating in a collaborative care DMHI to (1) characterize rates of trauma, PTS, and comorbid anxiety and depressive symptoms; (2) determine whether trauma exposure and elevated PTS symptoms impact the improvement of comorbid anxiety and depressive symptoms throughout participation in care; and (3) determine whether participation in collaborative care DMHI is linked to reductions in PTS symptoms. We found that trauma, PTS, and psychiatric comorbidity are common among youths participating in a DMHI, and comorbid PTS is associated with variations in rates of improvement for anxiety and depressive symptoms. Moreover, participation in a collaborative care DMHI is linked to improvements in PTS symptoms for most participants. These findings offer valuable preliminary insights into the clinical characteristics and sequelae among trauma-exposed youths participating in a DMHI.

Nearly 1 in 3 members participating in care for depressive, anxiety, or ADHD symptoms had experienced trauma. Many trauma-exposed youths reported symptoms of elevated PTS (n=119, 41%), and children with a traumatic event and elevated PTS had higher rates of elevated anxiety (n=87, 73%) and depressive symptoms (n=59, 50%) than children with no trauma. These observed rates of PTS, which are higher than previous estimates among trauma-exposed youths [13], paired with the high—albeit expected [31]—co-occurrence of anxiety and depression, highlight an acute need for DMHIs to provide both PTS screening and evidence-based treatment for youths with complex trauma-related symptomatology [32]. A slight majority (n=67, 56%) of those with elevated PTS were female, suggesting a limited role of sex in this sample’s PTS symptoms. Recent estimates suggest that more than twice as many women develop PTSD in adulthood as men [33], a discrepancy that remains largely consistent when controlling for trauma type [34]. However, these sex-based differences in PTSD prevalence may not arise until adolescence, during which pubertal changes catalyze developments in fear-related neurocognitive processing [35]. Incidence of sexual violence in adolescence may also contribute to sex-based differences in PTS: the majority of youths who experience sexual violence are between 12 and 17 years, and 82% of all sexual assault and abuse victims younger than 18 years are female [36]. Given the relatively young age of our sample, it is understandable that we did not identify such stark sex-based differences in PTS severity. Nevertheless, DMHIs should take into account sex-based differences in trauma and PTS risk factors when providing pediatric mental health care.

PTS symptom severity significantly impacted treatment response for anxiety. Youths with elevated PTS symptoms showed smaller reductions in anxiety symptoms compared to those without PTS symptoms. This is understandable, given the particularly close symptom overlap and etiology of PTS and anxiety [37,38]. Anxiety symptoms may be particularly related to PTSD in the form of anxiety sensitivity or the fear of anxiety-related sensations [39], with previous research suggesting that anxiety sensitivity is both retrospectively and prospectively related to PTSD severity [40,41]. PTS symptoms can also interfere with mental health treatment by exacerbating anxiety and other mental health symptoms, increasing feelings of overall distress, and decreasing receptivity to treatment [42,43]. It should be noted that youths with elevated PTS symptoms showed *larger* reductions in depressive symptoms; however, this effect was small and was not statistically significant. Given the small effect size paired with the limited sample, this finding requires additional study and replication before we interpret it further. In sum, these results suggest that among youths receiving digital mental health care for anxiety and depressive symptoms, screening for and consideration of PTS symptoms are crucial, as PTS may impact the timing and magnitude of treatment response. These findings also emphasize the importance of measurement-based care models such as the CoCM, that is, early and regular screening for PTS symptoms, which is a central aspect of the CoCM, is crucial to proactive treatment and long-term symptom improvement among trauma-exposed youths [32].

Although the intervention did not directly target PTS symptoms, most participants showed significant decreases in PTS symptoms throughout care. This finding likely points to the shared treatment targets (eg, emotion regulation [44]) and evidence-based methods (eg, CBT [15]) across PTS, anxiety, and depressive symptoms. Several DMHIs exist for the treatment of pediatric PTSD [18,45]; however, a recent review found that most are of poor quality and lack evidence- and measurement-based practice in the formation and implementation of the intervention [45]. As the usage of DMHIs for child mental health continues to increase, this study indicates that collaborative care DMHIs, which include high-quality evidence- and measurement-based care, are linked to secondary improvements in PTS symptoms via behavioral health care for depression, anxiety, and other mental health concerns (eg, ADHD). Taken together, these preliminary findings suggest that collaborative DMHIs may confer improvements in symptoms that are related to but outside the scope of treatment targets. Importantly, further experimental research is necessary to compare these effects with active and nonactive controls.

### Limitations and Future Directions

Although illuminating, these findings are limited by several notable factors. First, the retrospective nature of the study design limits us from drawing causal conclusions from our results. Further experimental research comparing the current DMHI with a randomized controlled group will offer more conclusive evidence for the effectiveness of the current intervention above and beyond another type of mental health treatment. Another consequence of the retrospective study design is that our results may be biased by participants self-selecting into care, given that nontreatment factors associated with mental health care use may also underlie symptom improvements (eg, family support, increased parental education, and perceived need [46]). Future studies should include a more rigorous study design with a randomized controlled group and data from long-term members.

This study did not address whether particular behavioral intervention methods—including coaching versus therapy and specific symptom target (eg, anxiety or depression)—may be more or less beneficial to mental health outcomes than other methods. Instead, we assessed outcomes associated with participation in the DMHI regardless of intervention methods. In future studies, identification of the behavioral interventions that are most beneficial to mental health outcomes in the context of DMHIs would greatly enhance the quality and efficacy of DMHIs in addressing PTS and comorbid anxiety and depression.

Given the relatively small sample size of children with trauma and elevated PTS symptoms, we were not able to gauge whether the nature of the participants’ trauma exposure (eg, type and timing) may have predicted their outcomes. A large body of research suggests that the development of PTS and comorbid psychiatric symptoms following trauma exposure is heavily correlated with the nature of the trauma [11,47]; as such, our analyses are missing a potentially significant covariate. While we reported the timing of trauma for members with an event, we could not assess timing as a potential covariate in further analyses. Future research should continue to assess whether the nature of a child’s exposure to a traumatic event may affect their outcomes and symptom trajectory within the context of a DMHI. Nonetheless, the high correlation between participants experiencing a traumatic event and exhibiting elevated PTS symptoms suggests that the trauma measure accurately reflected traumatic exposure.

### Conclusions

This study provides preliminary evidence for the frequency of trauma exposure and comorbid psychiatric symptoms, as well as variations in treatment response between trauma-exposed and nontrauma-exposed youths, among participants in a pediatric collaborative care DMHI. Youths with traumatic experiences may show increased psychiatric comorbidities and slower treatment responses than their peers with no history of trauma. These findings deliver compelling evidence that collaborative care DMHIs may be well-suited to address mental health symptoms in children with a history of trauma while also highlighting the critical need to assess symptoms of PTS in children seeking treatment.

## Appendix

Multimedia Appendix 1 Additional details regarding the categorization and analysis of race or ethnicity demographic question responses.

## Abbreviations

ADHD: attention-deficit/hyperactivity disorder
BCM: behavioral care manager
CBT: cognitive behavioral therapy
CoCM: collaborative care model
CPSS: Child PTSD Symptom Scale
DMHI: digital mental health intervention
DSM-5: Diagnostic and Statistical Manual of Mental Disorders, Fifth Edition
LRT: likelihood ratio test
PTS: posttraumatic stress
PTSD: posttraumatic stress disorder
