# Supplementary material for: Rates of Trauma Exposure and Posttraumatic Stress in a Pediatric Digital Mental Health Intervention: Retrospective Analysis of Associations With Anxiety and Depressive Symptom Improvement Over Time
Source: JMIR Pediatr Parent. 2024 Feb 27;7:e55560. doi: 10.2196/55560 (PMC10933721; doi:10.2196/55560)
Supplement: Multimedia Appendix 1 [file pediatrics_v7i1e55560_app1.docx]

# Multimedia Appendix 1

## Supplemental methods

To describe members’ race/ethnicity responses in a more succinct manner, race/ethnicity responses were grouped into larger categories for reporting, as follows. “Chinese”, “Vietnamese”, “Filipino”, “Korean”, “Japanese”, “Chamorro”, and “Other Pacific Islander” were reported as “Asian”. “Mexican, Mexican American, or Chicano”, “Puerto Rican”, “Cuban”, and “Other Hispanic/Latino” were reported as “Hispanic/Latino”. “American Indian or Alaska Native”, “Hawaiian/Pacific Islander”, “Other” and multi-select responses were reported as “Other or mulit-racial”. “White” and “Black or African American” were not grouped with other responses for reporting.
